# 

**Published:** 1859-08

**Authors:** 


### Contents of the Chicago Medical Journal, August, 1859.

ORIGINAL COMMUNICATIONS. PAGE.

ART. I. Two Cases of Lithotomy. Reported chiefly with reference to the Constitutional Treatment in connexion with Operations and Injuries. By David Prince, M.D., Jacksonville, Ill. .... 453
ART. II. Reduction of Dislocated Shoulder of six weeks' standing. By Ephraim Ingals, M.D., Prof. of Mat. Med. in Rush Med. College .... 463
ART. III. A New Method of Preventing Hemorrhage in Operations on the Lips. By Daniel Brainard, M.D., Surgeon of the U. S. Hospital at Chicago, etc .... 465
ART. IV. Prof. Donders' Mode of Assisting Vision in certain Opacities of the Cornea. By E. L. Holmes, M.D., of Chicago .... 466
ART. V. A Record of the Mortality of the City of Aurora, from July 1, 1858, to July 1, 1859. By L. H. Angell, M.D. .... 471
ART. VI. Operation for Tumor of the Throat, and on the Use of Scissors which crush the Tissues in operations for the removal of Tumors, etc. By Daniel Brainard, M.D., Prof. of Surgery in Rush Med. College, Chicago .... 473

PROCEEDINGS OF MEDICAL SOCIETIES.

Extracts from the Records of the Chicago Academy of Medical Sciences. S. C. Blake, M. D., Sec'y. 476
Medical Society in Northern Iowa. Medical Convention .... 479

EXTRACTS.

Clinical Report on Pneumonia .... 481
Retrospect on the Use of Raw Meat in the Diarrhœa of Weaned Children. By Dr. J. F. Weisse, Director of the Children's Hospital at St. Petersburgh .... 489
Epilepsy, in which Castration was performed .... 498

BOOK AND PAMPHLET NOTICES.

Anatomy, Descriptive and Surgical. By Henry Gray, F.R.S., Lecturer on Anatomy at St. George's Hospital. The Drawings by H. V. Carter, M.D., late demonstrator of Anatomy in St. George's Hospital. The Dissections jointly by the author and Dr. Carter. With 363 engravings on wood. Philadelphia: Blanchard & Lea. 1859 .... 498
Urinary Deposits, Diagnosis, Pathology, and Therapeutical Indications. By Golding Bird, M.D., F.R.S. Edited by Edmund Lloyd Birket, M.D., etc. A new American, from the fifth London edition. With 80 illustrations on wood. Philadelphia: Blanchard & Lea. 1859 .... 499
A Paper on the Management of the Shoulders in Examination of the Chest, including a New Physical Sign. Read before the New York Academy of Medicine. By John W. Corson, M. D., Lecturer on Diseases of the Chest, etc. (From the *New York Journal of Medicine* for March, 1859.) New York: H. Bailliere, 290 Broadway .... 499
Contributions to Operative Surgery and Surgical Pathology. By J. W. Carnochan, Professor of Surgery in the New York Medical College; Surgeon in chief to the State Emigrant Hospital, etc. With illustrations drawn from nature. Philadelphia: Lindsay & Blakiston. 1859 .... 500
Forty-second Annual Report on the State of the Asylum for the Relief of Persons deprived of the Use of their Reason. Published by direction of Contributors. March, 1859. Philadelphia .... 500
Transactions of the State Medical Society, of Michigan, at its Seventh Annual Meeting, held at Lansing, January 19th, 1859 .... 500
Constitution and By-Laws of the Chicago Academy of Medical Sciences. Adopted March, 1859. 500
A Lecture on Idiocy. By John M. Galt, M.D., Superintendent and Physician of the Eastern Lunatic Asylum of Virginia, at Williamsburg .... 500

MISCELLANEOUS MEDICAL INTELLIGENCE.

Proceedings of the Rock River Union Medical Society .... 502
An Inaugural Dissertation upon the Avulsion of the Arm and Scapula. By H. B. Horlbeck, of Charleston, S C. .... 504
On the Hysterotome for the Cure of Dysmenorrhea, invented By Dr. O. A. White, of Charleston, S. C. .... 505
Various Formulæ for the Gelatinization of Cod-Liver Oil .... 507
Cream as a Substitute for Cod-Liver Oil.—Chloroform .... 508
The Ecraseur.—Professional Habits in 1670.—Attendance of Medical Lectures in London..... 510
Preparatory School of Medicine.—Electricity as an Anæsthetic Agent in Dental Surgery...... 512
Evil of Smoking Tobacco .... 513

SURGICAL ITEMS.

New Medical Gazette.—Ovariotomy .... 514
Larrey, Father and Son .... 515

